# Medical Society of Louisville

**Published:** 1852-05

**Authors:** 


MEDICAL SOCIETY OF LOUISVILLE.

A society has been organized by the physicians of Louisville, of which we expect to make frequent mention. Its meetings will afford much material for our Journal. The right spirit is felt by its members, and the society can not fail to exert the happiest influence upon the profession in this city.

